# Exploring Protein-Based Carriers in Drug Delivery: A Review

**DOI:** 10.3390/pharmaceutics16091172

**Published:** 2024-09-05

**Authors:** Claudia Ferraro, Marco Dattilo, Francesco Patitucci, Sabrina Prete, Giuseppe Scopelliti, Ortensia Ilaria Parisi, Francesco Puoci

**Affiliations:** 1 Department of Pharmacy, Health and Nutritional Sciences, University of Calabria, 87036 Rende, CS, Italy; claudia.ferraro@unical.it (C.F.); marco.dattilo@unical.it (M.D.); francesco.patitucci@unical.it (F.P.); sabrina.prete@unical.it (S.P.); giuseppe.scopelliti29@gmail.com (G.S.); francesco.puoci@unical.it (F.P.); 2 Macrofarm s.r.l., c/o Department of Pharmacy, Health and Nutritional Sciences, University of Calabria, 87036 Rende, CS, Italy

**Keywords:** proteins, drug delivery, gelatin, albumin, collagen, zein, gliadin, silk proteins, soybean proteins

## Abstract

Drug delivery systems (DDSs) represent an emerging focus for many researchers and they are becoming progressively crucial in the development of new treatments. Great attention is given to all the challenges that a drug has to overcome during its journey across barriers and tissues and all the pharmacokinetics modulations that are needed in order to reach the targeting sites. The goal of these pathways is the delivery of drugs in a controlled way, optimizing their bioavailability and minimizing side effects. Recent innovations in DDSs include various nanotechnology-based approaches, such as nanoparticles, nanofibers and micelles, which provide effective targeted delivery and sustained release of therapeutics. In this context, protein-based drug delivery systems are gaining significant attention in the pharmaceutical field due to their potential to revolutionize targeted and efficient drug delivery. As natural biomolecules, proteins offer distinct advantages, including safety, biocompatibility and biodegradability, making them a fascinating alternative to synthetic polymers. Moreover, protein-based carriers, including those derived from gelatin, albumin, collagen, gliadin and silk proteins, demonstrate exceptional stability under physiological conditions, and they allow for controlled and sustained drug release, enhancing therapeutic efficacy. This review provides a comprehensive overview of the current trends, challenges, and future perspectives in protein-based drug delivery, focusing on the types of proteins adopted and the techniques that are being developed to enhance their functionality in terms of drug affinity and targeting capabilities, underscoring their potential to significantly impact modern therapeutics.

## 1. Introduction

Traditional drug administration methods, such as oral or intravenous ones, often suffer from significant limitations including poor bioavailability, rapid degradation, systemic toxicity and a lack of site-specific action. These challenges have encouraged the development of advanced drug delivery technologies for the loading and the controlled and specific release of pharmaceuticals to facilitate their safe absorption in specific areas of the body. This aim is reached by the preparation of drug delivery systems (DDSs) that play a crucial role in drugs’ administration, their targeting to specific sites, and their enhanced effectiveness in different therapies.

Throughout the advancements of drug delivery, different techniques have been utilized to enhance the adaptation of new therapeutic strategies, as demonstrated by the widespread adoption of controlled-release and extended-release (related to the chance of prolonging drugs half-life) systems. Moreover, an examination of the current state of the most employed methodologies (including drug surface modifications and their conjugation with specific ligands) indicates the constant need to find innovative approaches for facing important issues such as the presence of biological barriers or immune system reactions to immunogenicity-caused harmful effects [1].

**Figure 1:**
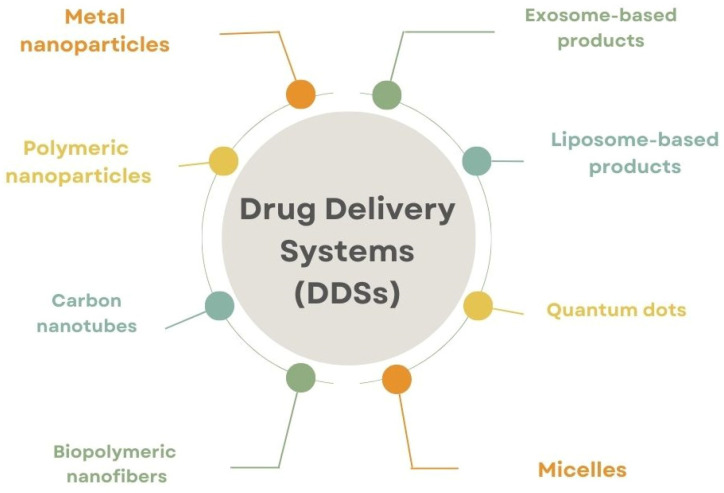
Some examples of different drug delivery systems (DDSs).

DDSs represent a gold mine, and they permit the efficient and non-invasive delivery of the therapeutic agents, concurrently protecting the loaded compounds from degradation or hostile environmental conditions. These systems include different and heterogeneous vehicles, for example: polymeric nanoparticles, metal nanoparticles, carbon nanotubes, biopolymeric nanofibers, micelles, quantum dots, liposome-based and exosome-based products, and many others [2], as shown in Figure 1. 

In order to overcome the existing challenges that are common to most DDSs, many authors have started to focus their attention on new approaches to enhance controlled and sustained drug release at targeted sites and to improve the water solubility of therapeutic agents. The latter property, indeed, is particularly significant because its resolution can lead to an optimal absorption of pharmaceuticals. 

A variety of natural and synthetic polymers have been employed in the preparation of different drug delivery systems, and many studies have also started to compare different materials adopted for this purpose. 

More specifically, natural polymers are organic substances present in the natural environment, and they represent suitable and adaptable vehicles for the creation of biomaterials used in both medical and ecological applications [3]. They offer many advantages such as water solubility, biocompatibility, biodegradability and non-toxicity [4,5]. Moreover, these materials show fewer side effects and are not as immunogenic as their synthetic counterparts; furthermore, they are inert and easily available. On the other hand, synthetic polymers can be described as polymers that are artificially synthetized in laboratories, often referred to as manufactured polymers [6]. They exhibit good conjugation abilities, but they also show many disadvantages, such as their less degradable nature and their role in causing inflammatory processes and immune response activation [7,8] (Table 1). 

Among the various materials explored for the fabrication of DDSs, proteins have attracted significant attention due to their unique properties including biocompatibility, biodegradability, and the ability to be engineered for specific functions. Therefore, interest in protein-based biopolymers for pharmaceuticals delivery has grown in recent years; proteins represent an advantageous frontier for the development of effective and new technology-based drug delivery systems. 

Proteins are naturally occurring macromolecules that play crucial roles in biological processes. They are composed of amino acids linked by peptide bonds and exhibit diverse structures, ranging from simple linear chains to complex three-dimensional conformations [9]. This structural versatility, combined with the ability to undergo post-translational modifications, makes proteins highly adaptable materials for DDSs [10]. Unlike synthetic polymers, which may elicit immune responses or require complex manufacturing processes, proteins are inherently biocompatible and can be produced in large quantities through recombinant DNA technology. Furthermore, the functional diversity of proteins allows for the design of DDSs with tailored properties, such as controlled release, targeting capabilities, and responsiveness to environmental stimuli.

Additionally, proteins are usually easily obtained from natural sources and quite simple to process under mild conditions, so they represent excellent candidates for the formulation of efficient DDSs. Proteins exhibit amphiphilic properties, facilitating effective interactions with solvents and a wide range of drugs. For example, protein nanoparticles can be synthetized under mild conditions, avoiding the need for organic solvents, and they are also able to establish covalent bonds with drugs and ligands. 

Proteins can be considered renewable sources extracted from plants, animals, humans, and other organisms. Plant proteins’ adoption and their modification technology represent a crucial field of biotechnology and pharmaceutical studies, aiming to tackle the growing demand for protein among humans. In particular, vegetal proteins have attracted great attention due to their interesting physicochemical and functional properties and their easy availability [11]. Numerous studies have highlighted the beneficial biological effects of plant proteins on biomedical research, such as their antidiabetic, anticancer, antioxidant, and kidney-protective properties. Additionally, they have been shown to lower cardiovascular and metabolic risk factors, and they also play a pivotal role in the regulation of lipid metabolism [12].

An interesting comparison can be drawn between natural protein-based DDSs and synthetic protein-based DDSs: natural biomolecules leverage the natural functions and properties of proteins to improve drug stability, enhance targeting, or facilitate controlled release, because of all the aforementioned features; their synthetic counterparts are designed for facilitating the targeted delivery of pharmaceuticals, and they represent an interesting alternative. Specifically, nanocarriers as drug delivery systems facilitate precise and regulated drug release, directing pharmaceuticals to specific cells or tissues while minimizing side effects [13]. 

In this context, the main goal of this work is to underline the most beneficial aspects of protein-based vehicles for drug loading and delivery and to focus on their principal challenges, in order to find innovative methodologies to overcome them. 

## 2. The Crucial Role of Proteins in Advancing Drug Delivery Systems

In recent years, protein-based drug delivery systems have emerged as a major research focus. The development of biopolymeric nanoformulations has emerged as a promising strategy for addressing various challenges associated with conventional DDSs. Microstructures and mostly nanocomposites made up of biopolymers have attracted great interest due to their ability to enhance drug loading capacity, bioavailability, and solubility and to provide sustained release mechanisms for poorly soluble pharmaceuticals. This represents an emerging frontier in pharmaceutical innovation and development [14]. 

**Figure 2:**
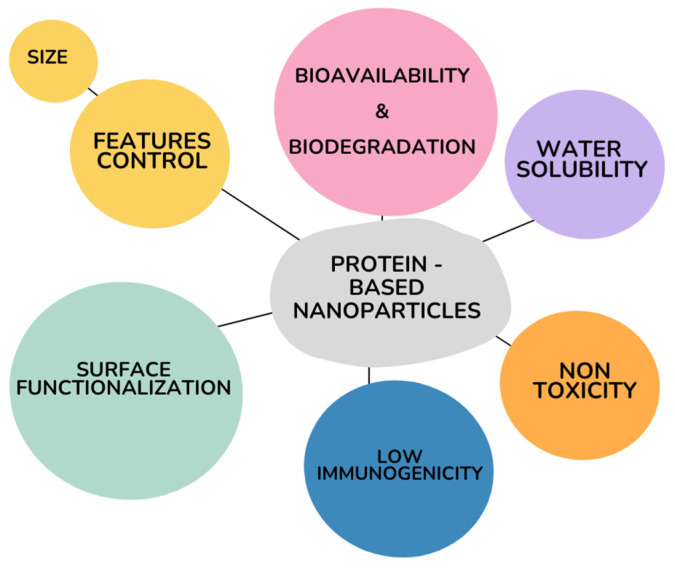
Advantages in the adoption of protein-based nanoparticles as drug delivery systems; data from [4].

There are numerous benefits to the use of proteins for the development of delivery nanostructures: these biomolecules are abundant in nature, their chemical features enable them to show optimal action at minimum dose, and they also facilitate processes of surface functionalization and coating in order to enhance the targeted delivery of many compounds. Specifically, protein nanoparticles offer several advantages as drug delivery systems (Figure 2), including biodegradability, low immunogenicity, and a straightforward control over particle size. The last aspect is crucial, because the control of this nanoparticles feature enables the rapid penetration of the drug-loaded compound into body tissues and fluids, a process that is much more challenging with larger, bulkier materials. For this reason, size control is considered one of the most important parameters not only for nanoparticles but in the synthesis of all DDSs [15]. 

In addition, compared to other colloidal carriers, protein nanoparticles exhibit a higher stability and an easier production process. Another interesting aspect related to such nanoparticles is that they are swiftly eliminated by macrophages [16]. Drug carriers are able to implement the targeting of active substances, and this is a positive aspect, which can support therapies and increase the accumulation of drug molecules in pathological areas [17]. One of the main advantages of using proteins in DDS development is represented by their ability to interact with biological systems in a highly specific manner. Proteins, indeed, can recognize and bind to specific receptors on the surface of target cells, enabling the targeted delivery of the therapeutic agents to diseased tissues. This targeted approach not only enhances the therapeutic index of the drug but also reduces side effects and systemic toxicity.

Moreover, protein-based drug delivery systems are able to prolong drugs’ systemic circulation time, and this is a useful tool for the development of new therapeutical approaches, particularly for drugs like cytokines and antibodies, which are characterized by high structural instability and short circulation time. Instead, the extension of nanocarriers’ blood circulation time enhances their capacity to accumulate in proximity of targeted tumor sites. 

Additionally, protein carriers can be modified with engineering techniques in order to express a specific feature. In the case of cancer, this may be accumulation in the area of the blood vessels that surround the tumor formation. 

The roles of the structure of drug carriers and their physicochemical properties are fundamental for maintaining their stability in the bloodstream and ensuring efficient drug delivery. The bioavailability of the carrier, indeed, is influenced by elements like size, shape, and surface coating with other substances [18]. In recent years, numerous delivery systems have been developed for pharmaceutical encapsulation and specific targets. However, effective approaches for achieving prolonged drug circulation times, such as PEGylation, remain quite scarce. As research progresses, increasing attention is being directed toward the prolonged circulation effects attributed to the physical or chemical characteristics of drug carriers. For example, carriers’ size is strongly associated with their circulation speed and tendency to aggregate in the bloodstream, which subsequently influences their in vivo permeability and distribution. Additionally, this important feature impacts immunogenicity and plasma half-life, thereby affecting both the circulation time of the vehicle and the therapeutic efficacy of the drug [19]. Another important aspect related to carriers’ size is represented by the correlated possibility of causing passive accumulation in tumor tissue through the enhanced permeability and retention effect. This effect arises from the abnormal vasculature developed within tumors, where particles smaller than 200 nm preferentially migrate into tumor tissues, while only smaller particles (less than 30 nm) are easily cleared. However, the optimal size range can vary significantly depending on the specific tumor [20].

In addition to the aforementioned carriers features, other physicochemical characteristics also deserve accurate investigation, such as different materials, which offer significant opportunities for designing innovative drug carriers. Extended circulation is achieved through the synergistic interplay of various characteristics rather than through isolated effects. A drug carrier optimized with advantageous physical and chemical attributes holds substantial potential for clinical application.

It is important to emphasize the key role played by proteins in their involvement in the construction of delivery systems: first of all, they are characterized by a high biocompatibility and a strong resistance against enzymatic degradation in the gastrointestinal tract; secondly, they can be engineered with many different techniques, in order to minimize the adverse effects mostly related to the phenomena of aggregation and dimers formation [21]. Proteins also offer the potential for developing DDSs that respond to specific stimuli, such as pH, temperature or the presence of specific enzymes. These stimuli-responsive systems are particularly useful in preparing smart carriers able to release their payloads in response to the microenvironment of diseased tissues, such as the tumor acidic environment or the presence of enzymes in inflamed tissues. 

**Figure 3:**
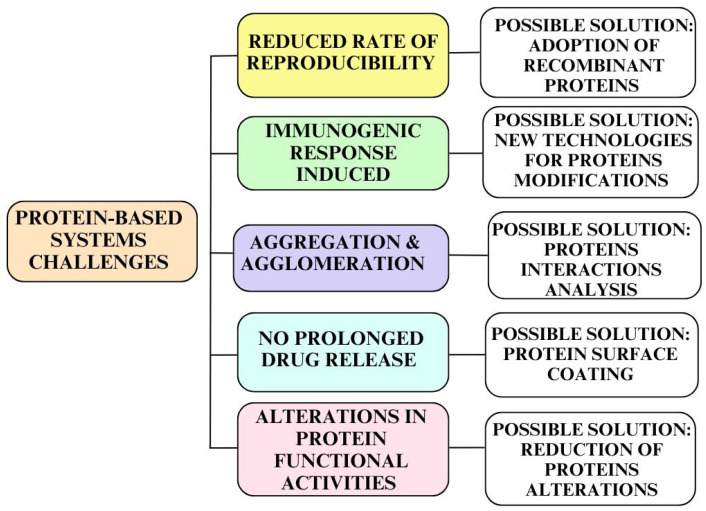
Challenges related to protein-based systems and possible solutions.

Despite the numerous benefits associated with protein-based vehicles adopted for drug delivery systems, there are certain challenges related to these natural polymers in the pharmaceutical and medical fields, as shown in Figure 3.

For example, proteins are heterogeneous mixtures of different size components [22], and this aspect can generate a reduced rate of reproducibility during the industrial processing of protein nanovehicles as drug delivery systems. A possible solution to this adversity is the production of recombinant proteins, the reason being that formulations that are uniform in size and have a specific molecular weight may be obtained. By engineering their structure, it is possible to attach various groups to their surface, as targeting or coating agents, and to control the rate of drug release. In this context, different types of proteins have been developed for drug delivery applications [23,24].

Another adverse aspect related to the entering of some protein-based carriers into the human body is a reduced but present immunogenic response, due to the presence of different origin proteins. An interesting strategy for facing this challenge and to prolonging pharmaceuticals’ half-life could be the adoption of new strategies for protein modifications: for example, PEG surface coating is able to mask immunogenic sites and to enlarge the drug’s hydrodynamic size, thus decreasing its renal clearance and extending its circulation half-life [25].

Furthermore, protein nanoparticles exhibit difficulties in the management of their molecular size due to possible toxic interactions with the living organism, and this aspect can negatively influence drug delivery and absorption. The presence of high free energies can lead to phenomena of aggregation and agglomeration, due to the poor physical stability of the systems [26]. An advantageous approach to preventing agglomerates’ formation is the adoption of different strategies to improve the solubility and stability of the involved proteins. One example is focusing on the analysis of their specific interactions, which are able to stabilize them, and promoting the use of surfactants or protective biopolymers [27]; a more straightforward method consists of keeping the protein concentration low, thereby increasing the sample volume, in order to reduce protein–protein interactions [28].

In addition, protein-based systems often present limitations in prolonged drug release efficiency; this happens because of the hydrophilic nature of proteins. When they absorb water in the body, their nanocarriers tend to swell and release drugs quickly. This aspect can negatively affect the therapeutic approach. For this reason, controlled drug delivery formulations are required, to be obtained using modified proteins. The latter aspect can be realized by the adoption of techniques such as antibody tagging or ligand attachment, in order to change proteins’ surfaces and to induce effective and long-lasting drug release [29]. 

Moreover, protein-based formulations show certain adverse aspects related to the introduction of structural modifications; the alteration of proteins’ structures can lead to the loss of their original features and functional integrity. In facing this challenge, we may find a possible solution in the reduction of the external modifications of the biomolecules, but other approaches need to be integrated [15]. 

New strategies are required in order to overcome these obstacles, and innovative approaches from different areas of research could be adopted for the achievement of “smarter” protein-based drug delivery carriers. Ongoing advancements in protein engineering, sophisticated drug delivery mechanisms, and production technologies will help overcome these challenges and improve the effectiveness and safety of protein-based carriers [30]. 

## 3. Protein-Based Drug Carriers

In the following section of the present review, attention will be focused on the design and development of protein-based delivery systems. Proteins, being versatile biopolymers, offer unique advantages in the field of drug delivery, such as the ability to form stable structures that can encapsulate and protect a wide range of therapeutic agents. In addition, their modifiable nature allows for the preparation of tailored carriers able to provide controlled release and targeted delivery (and, thus, enhanced therapeutic efficacy).

These systems can be designed in different forms, including nanoparticles, hydrogels and microspheres, which allow for specific challenges—such as improving the solubility of poorly water-soluble drugs, protecting sensitive molecules from degradation, and extending the circulation time of therapeutic agents in the body—to be addressed. 

**Figure 4:**
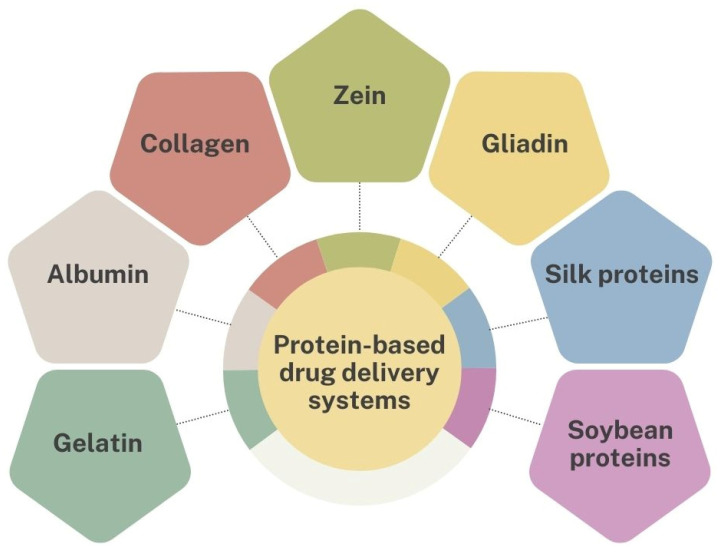
Examples of proteins used for the preparation of drug delivery systems.

In the upcoming sections, examples of DDSs utilizing various proteins, including gelatin, albumin, collagen, zein, gliadin, silk and soybean proteins (Figure 4), will be presented to illustrate the potential of these biopolymers in advanced therapeutic strategies.

### 3.1. Gelatin-Based Drug Carriers

Gelatins are extremely versatile natural proteins extracted from animal collagen (mostly from skin, hides, bones, scales and cartilage) using different procedures (acid, alkaline, and enzyme treatments; various extraction temperatures; and various extraction durations) that are commonly employed in the food industry and characterized by an eminent biocompatibility with human tissues. These proteins can be divided into different groups: mammalian gelatins (mostly bovine), fish gelatins (salmon, common carp, tilapia, tuna), and insect gelatins. Moreover, gelatin is a high-molecular-weight polyampholyte biomacromolecule; an interesting aspect of gelatin is that it contains cationic, anionic, and hydrophobic moieties in its molecular chain. Thus, due to chemical modifications, it offers a large number of readily accessible functional groups [31]. 

As a result of its gelling capacity, gelatin offers a great matrix for drug loading and delivery [32]. Furthermore, gelatin can play a pivotal role in the development of drug delivery systems when also combined with other macromolecules, leading to an improvement in anticancer therapies. 

For example, Prabha and Raj focused their attention on gelatin nanocomposites as valid tools for cisplatin delivery and controlled release, and this can become a promising innovation for cancer therapies. The aim of their study was the physical encapsulation of cisplatin—one of the most commonly adopted anticancer drugs—inside the nanocomposites made of Cassava starch acetate (CSA), polyethylene glycol (PEG) and gelatin (G). At first, there was the development of Cassava starch acetate–cisplatin nanorods, synthetized by nanoprecipitation with the adoption of NaOH/urea solution mixtures, followed by a dropwise add of CSA solution into an absolute ethanol solution; afterwards, the addition of PEG and G solutions, both prepared in water, led to the final nanocomposites. 

The drug release feature of these carriers was monitored, and a correlation between the speed of cisplatin release and the pH of the environment was observed. It was found out that acid conditions allow for better release of the drug, and moreover, the presence of the protein represents a good opportunity for drug delivery. Moreover, the selection of the physical encapsulation strategy allowed for the evaluation of gelatin as an effective drug delivery system, with benefits related to drug loading and its targeted release [33]. 

A recent study proposed the formulation of gelatin nanoparticles, produced by nanoprecipitation and solvent evaporation, for the loading and the targeted delivery of 5-aminosalicylic acid (5-ASA), a well-known drug adopted in therapies for the treatment of ulcerative colitis and which is poorly absorbed by the colon. These nanostructures were prepared in a water–base system, followed by the nanoprecipitation of gelatin into the nanoparticles, due to the use of an organic solvent. The nanocomposites were then coated with Eudragit-S100 enteric polymer, with a protective function against gastric pH-related environmental conditions. Gelatin’s role in this work was considerable, because it significantly helped in the development of an oral administered delivery system for the avoidance of the extremely acidic environment of the stomach. Furthermore, this system promoted the specific targeting of the inflamed colonic epithelium, with the enhancement in an anti-inflammatory response occurring due to the presence of 5-ASA drugs. The outcomes showed that the normal histology of the colon was significantly restored [34]. 

For the first time, Najafabadi et al. realized graphene oxide nanocarriers covered in gelatin and polyvinylpyrrolidone (PVP) for quercetin encapsulation. This flavonoid enhances the therapeutic efficacy of chemotherapy agents and, additionally, increases their toxicity, acting as a recommended antioxidant for cancer prevention. Extensive research has demonstrated that quercetin plays a crucial role in suppressing cancer cells in the areas of breast, colon, prostate, ovary, and lungs. Moreover, it exhibits powerful antiproliferative effects against cancer by sensitizing cancer cells to chemotherapy drugs and enhancing their efficiency. Quercetin also shows therapeutic properties, including antibacterial, anti-inflammatory, antidiabetic, and antiviral features, and it represents a benefit for the cardiovascular system. The main advantages of gelatin’s presence in these nanostructures are related to its principal roles in anticancer therapies: firstly, it contributes to the reduction of drug toxicity, and secondly, it ensures the prolonged retention and release of the drug in the tumor area. 

The preparation of the nanocomposite, made up of gelatin and PVP and coated with graphene oxide, consisted of the creation of a hydrogel and the loading of the drug into it. This choice represents an interesting strategy, because hydrogels are widely adopted and effective biomaterials for medical applications. Specifically, protein-based hydrogels can be developed as safe and targeted drug delivery systems. In addition to this, protein-based hydrogels are able to facilitate drug release in specific areas for a certain period of time, and this can be very useful for cancer therapies. 

After hydrogel formation, a double water–oil–water emulsion with the addition of bitter almond oil was formulated in order to obtain quercetin-loaded nanoparticles with a round shape for the control and the targeted release of the drug. It is interesting to note that quercetin is a hydrophobic compound, and it was previously dissolved in ethanol before being incapsulated into the nanoparticles. 

The nanocarriers exhibited a high encapsulation efficiency (87.5%) and a drug loading ability of 45%. Among the results, an interesting aspect is the potent cytotoxicity of the composites, which can cause the controlled apoptosis of cancer cells [35]. 

A different investigation proposed a novel pH-responsive drug delivery system, a nanocomposite made up of gelatin, chitosan (a pH-sensitive biopolymer), and carbon quantum dots for curcumin delivery. The integration of quantum dots (made via a hydrothermal process) into the physically crosslinked hydrogel (which was water-soluble and composed of gelatin and chitosan) was achieved using a water–oil–water double-emulsion technique (W/O/W). The choice of gelatin in combination with chitosan was particularly convenient; the presence of the protein increased the possibility of making a biodegradable hydrogel characterized by pH-dependent solubility. This aspect is useful in drug controlled release. 

Additionally, the creation of this innovative nanosystem was able to enhance curcumin’s half-life. Based on the obtained results, this work suggested that the obtained nanocomposites are biocompatible and promising nanocarriers for the enhancement of curcumin delivery in different therapies; moreover, they exhibited cytotoxic effects against specific cancer cell lines [36]. 

Jaberifard et al. developed an alternative approach for the delivery of carvedilol (a poorly water-soluble drug used in the treatment of hypertension and coronary artery pathologies); initially, the drug was loaded into halloysite nanotubes. Then, the system was prepared using nanotubes and gelatin microparticles and a water-in-oil emulsion (w/o) protocol, with the use of glutaraldehyde solution as crosslinker agent. Halloysite nanotubes exhibited a negatively charged external surface and an internal layer covered by positive charges; these features promoted drug loading and absorption due to the formation of hydrogen bonds and electrostatic interactions. Additionally, nanotubes were enriched with gelatin due to its outstanding pharmacological features and ease of surface modification. Studies on drug release have demonstrated that gelatin offers effective shielding from the gastric acidic environment. Controlled drug release within the intestinal tract and enhanced administration stability over an extended period using microparticles were also noted and attributed to the pH-sensitive properties of gelatin. Based on these findings, the formulated and insoluble microparticles were presented as a suitable and interesting oral drug delivery system for the controlled release of different pharmaceuticals [37]. 

Numerous studies have highlighted the versatility and effectiveness of using gelatin nanoparticles (GNPs) as drug delivery systems [38]. For instance, a study from 2002 detailed the development of biodegradable hydrophilic and gelatin NPs for the loading of different concentrations of methotrexate drugs (often used in anticancer treatments), adopting a solvent evaporation method with a single water-in-oil emulsion. This procedure was enriched by the use of glutaraldehyde as a crosslinking agent.

The observed parameters related to the mechanism of drug release were found to be optimal, so, according to data, gelatin nanostructures were able to enhance stimuli-responsive drug release [39]. 

Zhong et al. focused their attention on the use of gelatins as emulsifiers for oil-in-water emulsions. An emulsion typically refers to a blended colloidal system that overcomes the immiscibility of water and oil by the dispersion of one phase as droplets within the other phase [40]. The emulsifying properties of gelatin are influenced by its sources, extraction methods, and molecular weights. The authors also underlined the positive aspects of making physical, chemical, and enzymatic modifications to gelatin in order to obtain stabilized emulsions. In this regard, the interaction of gelatin with various molecules—as different surfactants—at the oil/water interface represents an effective method of stabilizing emulsions. All the aforementioned properties make gelatin a versatile component in the production of stable and effective drug delivery systems [41]. 

Furthermore, the formation of nanocomplexes containing gelatin as a good emulsifier has continued to capture researchers’ interest. Wang et al., for example, developed insoluble gelatin type B/chitosan nanoparticles, which were found to be good Pickering emulsifiers (in Pickering emulsions, solid or colloidal particles are adopted as stabilizers instead of surfactants). A study of the polysaccharide–protein complex was conducted in order to elucidate the insolubility in the preparation of the oil/water emulsions at different pH levels. In fact, this work underlined the effects of pH changes and storage time on the formation of such nanocomposites and also the pivotal role played by gelatin in combination with chitosan [42]. Another study showed the preparation of gelatin/glucomannan (a neutral polysaccharide characterized by a gel similar structure and a good water solubility)/tannic acid nanocomplexes: these nanostructures were realized by the particle self-assembly procedure, and they were thought to be tools for stabilizing Pickering emulsions. The outcomes showed positive effects in that sense [43]. 

Leiva-Vega et al. created an original nanosystem for the encapsulation of curcumin dissolved in coconut oil: the drug was loaded into a multilayer emulsion made up of gelatin as the primary layer, gum arabic as the secondary layer, and tannic acid as the tertiary layer. This procedure was carried out via a layer-by-layer deposition technique, and it was refined by the use of coconut oil as a stabilizer in the primary emulsion because of its good bioavailability in oil–water emulsions for the transport of lipophilic compounds. 

The gelatin concentration proportionally influenced the stability of the primary emulsion. This multilayer approach enhanced the prolonged preservation of the antioxidant activity of the emulsified curcumin [44]. 

Another study focused on achieving effective Pickering emulsions by incorporating additional hydrophobic amino groups into gelatin nanoparticles, resulting in new forms of aminated-gelatin nanoparticles. In these nanoformulations, gelatin was modified with ethylenediamine using the Morimoto method [45] to obtain an aminated form of the protein, which was then used to prepare the nanoparticles. 

The nanoparticles exhibited increased surface charge, higher hydrophobicity, and enhanced flexibility compared to native gelatin nanoparticles. Furthermore, emulsions stabilized by aminated gelatin nanoparticles outperformed those stabilized by native gelatin nanoparticles, confirming the benefits of this chemical modification of the protein [46].

Focusing on the different technologies available for the preparation of nanoparticles, the nanoprecipitation technique (using water and ethanol as the solvent and nonsolvent phase) offers several benefits, being simple, rapid, and easily executable. Nanoparticle formation happens immediately, and this is an important element that makes the process effective and commonly adopted. 

In a recent work, gelatin nanoparticles were synthesized using the nanoprecipitation technique. The research focused on examining the loading efficiency and the simultaneous delivery of two interesting drugs: tizanidine hydrochloride (5-chloro-N-(4,5-dihydro-1H-imidazol-2-yl)-2,1,3-benzothiadiazol-4 amine hydrochloride), a muscle relaxant, and gatifloxacin (1-cyclopropyl-6-fluoro-8-methoxy-7-(3-methylpiperazin-1-yl)-4-oxo-quinoline-3-carboxylic acid), an antibiotic utilized in various therapies. The results highlighted the role of gelatin into the formulation; furthermore, the drug release studies showed that the release profiles of the two pharmaceuticals were comparable and demonstrated enhanced drug delivery [47]. 

Das et al. synthesized GNPs by combining gelatin with folic acid; this ligand is quite interesting, and it can be easily combined with an assortment of nanocarriers (like linear and branched polymers, polymeric micelles, dendrimers, nanotubes, and nanosheets and liposomes) thanks to its γ-carboxylate group. In this study, folate was conjugated to the gelatin surface in order to overcome the major limitations of shortened circulation half-life. Moreover, the conjugation was followed by the nanoprecipitation technique in the presence of a hydrophilic polymer (polysorbate 80). The study focused on the encapsulation of the chemotherapeutic drug irinotecan, and the results showed that the presence of folic acid has an influence on the final yield and loading efficiency [48]. 

An American study detailed the development of an interesting protocol (based on the two-step desolvation method) for the preparation of ultra-small gelatin nanoparticles—GNPs—(10 nm), small GNPs (50 nm), and medium GNPs (200 nm). This technique consisted of a first desolvation step, with the presence of acetone for the precipitation of the high-molecular-weight part of gelatin, and a second desolvation step with the involvement of a nanoprecipitant solution. An important element is the adding of trypolyphosphate as an anionic crosslinker, which led to the formation of ultra-small gelatin nanoparticles. 

The research was focused on the encapsulation of doxorubicin, iodixanol and cisplatin, and the GNPs of 10 nm exhibited superior penetration if compared to the larger ones. Additionally, strategies were developed to encapsulate drugs or contrast agents, and they can be employed for advanced biomedical applications [49]. 

All the presented studies are reported in Table 2.

### 3.2. Albumin-Based Drug Carriers

Albumin is a water-soluble globular protein found in blood plasma. It is the most prevalent protein in the human bloodstream, and it is produced in the liver, where hepatocytes translate it from a single gene as preproalbumin. This pre-form is then moved to the endoplasmic reticulum, where a serine protease cleaves the N-terminal prepropeptide. Following this, the protein is transported to the Golgi apparatus and then released into the bloodstream as a basic protein [50]. 

**Figure 5:**
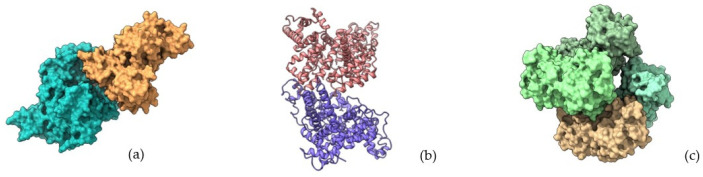
(**a**) Bovine serum albumin (BSA), (**b**) human serum albumin (HSA), and (**c**) ovalbumin (OVA) structures.

Albumin-based drug delivery systems have appeared promising therapeutics in the diagnosis and treatment of cancers. Bovine serum albumin (BSA), human serum albumin (HSA), and ovalbumin (OVA) have been employed as nanocarriers for the delivery of drugs, antibiotics, and peptides, as shown in Figure 5.

Jalali et al. focused their attention on the synthesis of BSA/oxidized arabic gum nanoparticles (its oxidation was carried out with sodium metaperiodate, and it was used as an efficient, green, and biodegradable crosslinker), with the use of the desolvation method. 

Their study was focused on the loading of piperine, an alkaloid from black pepper, and reports and characterizations indicated that the encapsulation efficiency improved proportionally with the increase in the amount of crosslinker. There was also a computational part of the analysis in the form of an in silico molecular docking of the interactions between BSA/OGA complex and piperine, and it showed that there was good binding affinity [51].

Ma et al. worked on the use of folic acid and grafted BSA complexed together as stabilizers for the preparation of graphene oxide (GO)-based drug carrier systems and the delivery of doxorubicin. The second step was the formation of FA-BSA graphene oxide nanocomplexes, followed by doxorubicin loading by mixing. The results showed that the nanohybrids could specifically deliver the drug to folate receptor-rich cells (MCF-7 cells), reaching a high rate of targeted drug delivery. This was the first instance in which an FA-grafted BSA molecule was used as a targeting agent to disperse graphene oxide for drug delivery, and the presence of BSA represented a significant advantage [52]. 

Another interesting study showed an innovative method for the delivery of pterostilbene (3,5-dimethoxy-4′-hydroxystilbene), a phytoalexin derivative from resveratrol which is characterized by various biological activities, such as hypolipidemic, antioxidant, antidiabetic, and anticancer effects. Its applications and bioavailability are significantly restricted by its poor water solubility and stability. Among many different strategies, the use of ethoniosomes represents a promising tool for drug delivery. Ethoniosomes are particular kinds of niosomes (nanocarriers formed through the self-assembly of nonionic surfactants in an aqueous environment, leading to closed bilayer formations, as initially investigated by researchers at L’Oréal—Clichy, France—for cosmetic use). Since then, niosomes have been widely studied for various applications across different fields, including pharmaceuticals and food sciences [53,54]. Ethoniosomes are more flexible forms of niosomes, which contain ethanol and a low quantity of cholesterol. In this study, ethoniosomes have been developed, adopting the proethoniosomes formulation method, which consists of the building of pro-vesicles that can be converted into niosomes upon hydration. The formation of ethoniosomes was enriched with the coating of folic acid conjugated BSA and, based on the findings, these vesicles showed potential as a successful targeted drug delivery system for lung cancer therapy [55].

Another recent work detailed a drug delivery system, designed as follows. First, the Fe^3+^–BSA nanocomplex was formed. Next was the loading of doxorubicin with the desolvation–crosslinking method (a well-developed technology for preparing protein nanoparticles) and the use of indocyanine green, which is commonly employed in photodynamic and photothermal therapies (often coupled with chemotherapy). The nanoparticle surface was grafted with folic acid, and this element considerably improved the ability of the nanocomposites to specifically target tumors [56].

All the presented studies are reported in Table 3.

### 3.3. Collagen-Based Drug Carriers

Collagen is a key structural protein that is abundant in the human body, mostly in connective tissues like skin, bones, tendons, and ligaments. It imparts strength and support due to its unique triple-helix structure, which provides tensile strength and stability to tissues. Collagen also is responsible for cell adhesion, proliferation and differentiation and it is crucial for maintaining skin elasticity, promoting wound healing, and supporting joint and bone function.

In drug delivery, collagen is studied and adopted for its biocompatibility, biodegradability, and low immunogenicity. It represents an effective carrier for various therapeutic agents, enabling targeted release of drugs and enhancing the efficacy of treatments while minimizing side effects. This makes collagen a very promising material in developing advanced drug delivery mechanisms. 

Qi et al. described the preparation of collagen—(poly 3-acrylamidophenylboronic acid, PAPBA) nanoparticles for the loading of doxorubicin and its study in ovarian cancer. The encapsulation efficiency was very high; moreover, the very good release test results indicated that the nanoparticles exhibited a high drug release rate [57]. 

A fascinating work described the choice of type 1 collagen (extracted from the skin of *Carassius carassius*, commonly known as the crucian carp) for the preparation of hydrogels using the plastic compression technique to increase the mechanical features of the products, making them useful tools in wound-healing therapies. The study also evaluated the encapsulation efficiency and targeted release of luteolin (3′,4′,5,7-tetrahydroxyflavone), a natural flavonoid with numerous therapeutic properties. The results indicated an improvement in the wound healing process, suggesting a promising innovation in wound healing therapies and management [58].

Yue et al. synthetized cellulose nanofibrils, which are widely adopted in biomedical studies due to their interesting features, such as their ease of surface modification. They also prepared collagen aerogels through a self-assembly treatment followed by freeze-drying in order to explore their potential as drug delivery systems with advantageous characteristics. The authors developed a fascinating structure made up of cellulose nanofibrils and collagen aerogel in order to induce the self-assembly of collagen into the nanofibril network. The final nanocomposite showed a pH-responsive feature and a strong structural stability. Although preliminary studies were conducted, including analysis of the release of 5-fluorouracil as a model drug, further research is needed to fully explore its potential as a drug delivery system [59]. 

Zhang et al. fabricated porous microspheres made of a formulation of collagen and bacterial cellulose. This combination helps protect the integrity of the collagen, shielding the protein from protease activity and thermal fluctuations. The microspheres were built using a template method followed by an inverse suspension regeneration, and they were studied for their ability to load, absorb, and release BSA, a model protein. This study represents a first stage of application of controlled drug delivery and release by collagen-based microspheres, and further studies are needed to validate these preliminary results [60]. 

Rathore et al. investigated the role of silymarin (a polyphenolic flavonoid extracted from milk thistle, known for its antioxidant properties)-loaded collagen nanoparticles as a brain-targeting drug delivery system. This study showed the enhanced therapeutic effect of silymarin due to the nanocomposite formulation, primarily due to the encapsulation of the drug. This advancement suggests the potential for innovative therapeutic approaches, using collagen nanoparticles, to treating brain diseases [61]. 

All the presented studies are reported in Table 4.

### 3.4. Zein-Based Drug Carriers

Zein is a prolamin-rich protein, which was first isolated from whole white maize and named by John Gorham in 1821. It shows a high quantity of hydrophobic non-polar amino acids, which improves its hydrophobic drug loading ability and also promotes self-assembly into stable nanoparticles. 

Due to its self-assembly, zein has been extensively investigated for the encapsulation of bioactive compounds. It is a very versatile, hydrophobic, and water-insoluble (but soluble in hydroalcoholic solutions) protein, and it is characterized by some interesting features, as low immunogenicity, biodegradability, biocompatibility, and gastrointestinal resistance. Because of all these advantages, zein is commonly selected in research areas focused on enhancing oral drug bioavailability and targeted drug delivery. The clinical implementation of drug-loaded zein-based carriers still represents a challenge, due to the limited amount of research data available [62]. 

Wang et al. tried the encapsulation of doxorubicin into zein nanoparticles prepared using the phase separation method. In comparison with the aspecific release system of doxorubicin, the nanoparticles demonstrated slower drug release with normal extracellular pH conditions and faster drug discharge in acidic pH conditions; this suggests that zein nanoparticles are able to extend the drug’s circulation time in the bloodstream and also to improve targeted cytotoxicity toward specific tumor cells. The obtained optimistic results suggested that nano-encapsulation using zein could be an effective drug delivery system for cancer chemotherapy [63]. 

Yang et al. prepared zein nanoparticles for the loading of maytansine (a potent tubulin polymerization inhibitor typified by poor water solubility and toxic side effects) and in order to check the nanocomposites’ effectiveness as drug vehicles for the treatment of non-small cell lung cancer. Cell and animal experimental results showed that the nanoparticles exhibit strong anti-tumor cell activity in both in vitro and in vivo studies [64].

A recent study documented the realization of zein nanoparticles loaded with luteolin (3′,4′,5,7-Tetrahydroxyflavone). One of the challenging aspects of zein nanoparticles is that due to the hydrophobic surface of zein and its associated chemical characteristics, these formulations are not very stable and tend to aggregate. For this reason, it is preferable to use surfactants or biopolymers to coat the nanocomplexes; in this study, the authors chose sodium caseinate (a soluble mix of casein proteins), and the results showed that their presence stabilized the nanoparticles and incremented luteolin loading and its delivery [65]. 

Rashed et al. proposed a new integration of gene therapy and nanocarriers as a promising tool in therapies for hepatocellular carcinoma; they proposed the formulation of zein nanoparticles as a new delivery system for PTEN (phosphatase and tensin homolog deleted from chromosome ten) and TRAIL (TNF-related apoptosis-inducing ligand) genes, which are two oncosuppressors. The results showed that PTEN and TRAIL inhibited the proliferation of liver tumor cell lines, and their targeted delivery was enhanced by the use of zein nanoparticles [66]. 

The use of biopolymeric nanofibers for the loading and delivery of different substances for various applications is a topic of interest for many researchers. Furthermore, for biomedical applications, the preparation of nanofibers mainly occurs with the electrospinning technique, which is able to create a large area for drug loading and delivery. Often, the nanovehicles are coated or tailored with other molecules for the enhancement of specific functions. 

Zein-based nanofibers can be categorized into four classes according to their structural features: pure nanofibers, hybrid nanofibers, crosslinked nanofibers and core–shell nanofibers [67]. 

Wongsasulak et al. developed zein nanofibers also made of chitosan and polyethylene oxide (PEO) for the loading of alpha-tocopherol; the nanocomposites showed optimal mucoadhesive properties and they appeared as potential vehicles for compounds’ delivery, particularly in the gastrointestinal tract [68]. 

A recent study focused on the preparation of zein nanofibers with the incorporation of tungsten oxide (this choice of metal oxide nanostructures for potential cancer therapy is due to their ability to cause various effects, including DNA damage). The authors characterized them, analyzing their possible therapeutic role against melanoma, and found out that these nanofibers represent a possible and safe candidate for anticancer therapies [69]. 

All the presented studies are reported in Table 5.

### 3.5. Gliadin-Based Drug Carriers

Gliadins represent a group of water-insoluble but alcohol-soluble prolamin proteins extracted from gluten (the source of which is wheat and numerous other cereals) using 70% ethanol. Rich in neutral and hydrophobic amino acids, such as glutamine and proline, gliadins can be classified, considering their electrophoretic mobility in acidic conditions, as α- and β-gliadins (from 28 to 35 kDa), or γ- and ω-gliadins (from 35–40 to 70 kDa) [70]. 

Due to their hydrophobicity and low solubility in aqueous conditions, gliadins are particularly suitable for the loading of poorly water-soluble drugs through a desolvation process. 

Gliadin proteins show favorable interactions with biological membranes; moreover, they exhibit interesting emulsifying and mucoadhesive properties, which could be very useful for the oral delivery of lipophilic drugs. 

Gliadin nanoparticles also represent an efficient drug delivery strategy for drug targeting in the upper region of gastrointestinal tract. 

In a recent work, Fresta et al. synthetized gliadin nanoparticles (by nanoprecipitation) with a coating of polyoxyethylene (2) oleyl ether for the loading and the delivery of doxorubicin hydrochloride. The obtained outcomes draw attention to the possible use of gliadin nanocomposites as optimal carriers for antitumor compounds [71].

Another study described the preparation of gliadin nanoparticles functionalized with hyaluronic acid for the targeted delivery of usnic acid (a natural antineoplastic drug) to breast cancer cells, particularly to CD44 receptors. Further research is needed to assess the efficacy of these proposed formulations in antitumor therapies, but the preliminary results are very promising [72].

Huang et al. developed some hybrid nanoparticles made of gliadin and silver to obtain antibacterial nanostructures that could be useful in counteracting infections and diseases. The presence of gliadin is fundamental because silver nanoparticles have some limitations. For example, while these ultrasmall nanostructures exhibit higher antibacterial activity than larger ones, they tend to be reactive and unstable, often forming aggregates and leading to oxidation phenomena. In order to overcome these drawbacks, engineering techniques—applied to the nanocomposites and with the involvement of natural macromolecules—may be adopted. One interesting approach explored in this work consisted of the use of protein nanoparticles to form nanoplatforms, which included silver nanoparticles, for the building of a protein-based porous material. This material was designed to encapsulate the silver nanoparticles and enhance their therapeutic activity. The results showed that the obtained formulations reached a high stability in physiological solutions; moreover, they exhibited fast and controlled release of silver ions. This good performance and proven ability to inhibit the growth of some tested bacteria represent a good starting point for further speculations about this system [73]. 

Wang et al. studied the therapeutical role of wheat gliadin hydrolysates, obtained by enzymatic hydrolysis, in the preparation of nanomicelles for the loading and encapsulation of naringin (a natural flavonoid characterized by antioxidant, anti-inflammatory, and antineoplastic features, but which has low water solubility). Some of the outcomes exhibited the increased bioavailability of the drug, enhanced by the leading role of gliadin that, due to the hydrolytic approach, was able to enhance the exposure of hydrophobic or hydrophilic regions within its own structure, thereby improving its solubility and amphipathic properties [74].

Marcano et al. produced a formulation of gliadin/casein nanoparticles due to the recognized role of caseins (which were adopted for the surface coating of the nanoparticles) as stabilizers and optimizers of gliadin nanoparticles’ dispersion in water. These nanostructures were developed for the loading and targeted delivery of amphotericin B, a well-known drug mostly adopted for fungal infections. The nanoparticles were synthetized with the antisolvent precipitation methodology, and they were able to show good stability in simulated gastrointestinal fluids, with optimal drug release. Moreover, the choice of gliadin was useful because its amino acid composition facilitated interactions with the gastrointestinal mucosa, thanks to the formation of hydrophobic bonds; this then led to an improvement in mucoadhesion, which has great utility in the production of oral drug delivery systems [75]. 

All the presented studies are reported in Table 6.

### 3.6. Silk Protein-Based Drug Carriers

Silk proteins obtained from various silkworm species (such as *B. mori* for mulberry silk or orb-weaving spiders for non-mulberry silk) display many differences in their structure and properties. These biopolymers are employed in drug delivery and biomedical applications because of their distinctive mechanical and physicochemical features, including biocompatibility, gradual biodegradability, and self-assembly abilities. The most abundant silk proteins, which are commonly used in pharmaceutical studies, are fibroin and sericin. 

Silk sericin is a water-soluble protein derived from silk, specifically produced by the silkworm *Bombyx mori* and characterized by a hydrophilic nature and versatile biological activity (for example, it can have both antioxidant and anti-inflammatory effects). 

**Figure 6:**
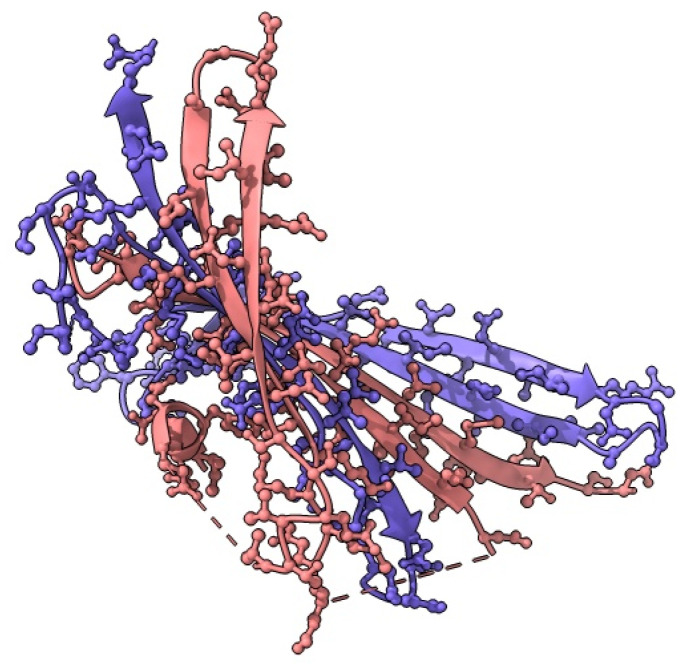
Silk fibroin structure.

Silk fibroin derived from silkworm cocoons (*B. mori*) is the most widely utilized silk for controlled drug and protein delivery, as shown in Figure 6.

Lately, the synthesis of fibroin nanoparticles (FNPs) for different biomedical applications has been extensively researched. Due to their chemical versatility, FNPs can incorporate a wide range of therapeutic substances, including molecules of different size, proteins, and enzymes [76]. Different studies highlight the effectiveness of these nanosystems in drug delivery. For example, Lozano-Perez et al. studied the enhancing effects of FNPs on the encapsulation, adsorption, and targeted delivery of quercetin by monitoring its release in the gastrointestinal tract. These nanostructures were synthetized using the desolvation technique, and the loading of quercetin was accomplished with a simple incubation. The results showed that these nanocomposites are able to protect drugs against degradation in the gastrointestinal area This characteristic suggests a potential role for them in therapeutical approaches and the development of non-invasive nanoplatforms [77].

Gupta et al. focused their attention on silk fibroin blended with chitosan, forming non-covalent complexes. These complexes were then used for the functionalization of curcumin-loaded nanoparticles, which were synthesized using the capillary microdot method. The goal was to enhance the bioavailability of curcumin and its anticancer role through its specific release at tumor sites. Some interesting outcomes related to curcumin delivery were achieved by both silk fibroin nanoparticles and silk fibroin–chitosan nanoparticles; the coating of silk fibroin nanostructures could be a potential tool for the development of innovative treatments and therapies for tumors and numerous other diseases [78].

A recent study described the development of a silk fibroin/casein blend for facilitating drug release. This blend involved the use of pure silk fibroin electrospun nanofibers and the synthesis of both silk nanostructures and silk/casein nanostructures, employing the electrospinning technique. These nanocomposites were able to ensure the loading and the targeted release of diclofenac sodium salt (an anti-inflammatory drug). Tests checking the nanovehicles’ cytotoxicity and biocompatibility were conducted on fibroblasts, and the results indicated that the combination of silk fibroin and casein was more successful in improving the delivery and targeted release of drugs [79].

Tallian et al. investigated the therapeutical possibilities of silk fibroin–human serum albumin nanocapsules, focusing their attention on their stability. These nanostructures were designed with an interesting mechanism of pH-responsive drug delivery and targeted release, for the potential treatment of inflammatory diseases. The release of nanocapsules’ drug content was allowed only in an acidic environment, due to the fact that inflammation processes lead to a decrease in pH levels in lysosomes. For this reason, the authors focused on this mechanism for the selective release of nanocapsules’ content only in proximity to inflamed tissues, with the adoption of methotrexate as model drug, and this study represents an innovative approach to the treatment of inflammation [80]. 

Numerous works have also underlined the important role of silk sericin-based nanovehicles for the enhancement of drug loading and its targeted release. For example, Saraf et al. synthetized silk sericin nanoparticles (adopting a desolvation technique with the use of genipin as a crosslinker to optimize the process) for the loading and the delivery of atorvastatin, a synthetic form of statin which is commonly used in different cancer treatments, such as breast cancer [81], gastrointestinal carcinoma [82], and pancreatic cancer therapies [83]. This work showed that the obtained nanoparticles were biocompatible and, moreover, they exhibited good control of drug release, representing a promising way of improving the therapeutic role of atorvastatin [84]. 

Suktham et al. developed sericin nanoparticles and chose the adoption of Pluronic F-68 (a surfactant) as a stabilizer to enhance the loading of resveratrol (*trans*-3,5,4′-trihydroxy-stilbene), a polyphenolic compound known for its anticancer properties. This study presented the obtained nanostructures’ ability to control and increase resveratrol encapsulation and its targeted release compared with other delivery systems. Moreover, their presence and action inhibited the growth of colorectal adenocarcinoma cells, and this may lead to the development of new therapeutical approaches for different forms of cancer [85]. 

An interesting study described the preparation of sericin/poly(ethylcyanoacrylate) nanospheres to explore the effects of the combination of poly(alkylcyanoacrylates) with mucoadhesive proteins for the building of novel and effective drug delivery systems. The synthesis of the nanostructures was realized with interfacial polymerization in aqueous media. Moreover, the nanospheres were tested for enhanced fenofibrate (a lipophilic drug used for cholesterol diseases) delivery and oral bioavailability, with a specific focus on its targeted release into the gastrointestinal area. The in vivo and in vitro results underlined an improvement in the therapeutical absorption in proximity to the gastrointestinal mucosa, and this could lead to the development of nanospheres as delivery vehicles for drugs characterized by poor water solubility [86]. 

A different work adopted silk sericin for the preparation of a bioconjugate obtained by free radical grafting of sunitinib (a synthetic drug commonly used in many anticancer therapies) on sericin protein, using hydrogen peroxide and L-ascorbic acid as a redox pair. The evaluation of in vitro gastrointestinal availability showed increased transport of the drug due to the features of the conjugate that led to an increase in the drug’s water solubility. The obtained results could lead to the introduction of an innovative method, based on the use and the modification of silk sericin, for the improvement of drugs’ bioavailability [87]. 

All the presented studies are reported in Table 7.

### 3.7. Soybean Protein-Based Drug Carriers

In recent years, natural polymeric hydrogels have demonstrated significant potential as drug delivery systems due to their distinctive properties, including biodegradability, biocompatibility, and non-toxicity. Great focus has been placed on soybean proteins because of their interesting and promising features [88]. 

**Figure 7:**
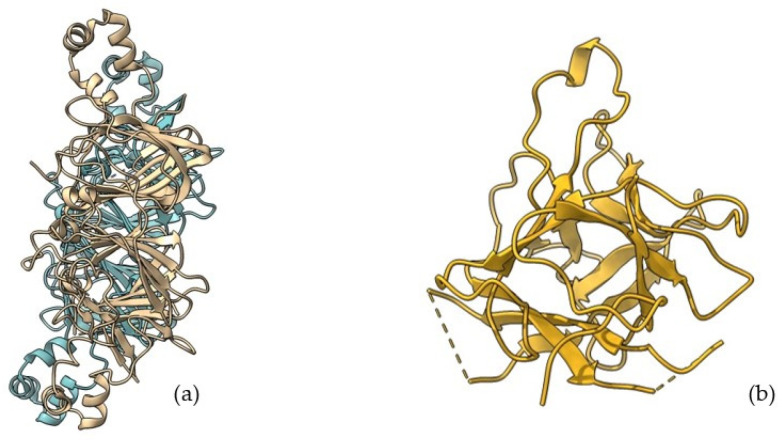
Soybean protein structures: (**a**) crystal structure of glycinin, from soybean proteins; (**b**) trypsin inhibitor from soybean.

Soybean proteins have high nutritional value and numerous functional properties, such as emulsification, foamability, and gelation. They can be extensively utilized as food supplements, emulsifiers, and in pharmaceutical products, as shown in Figure 7.

A recent study described the synthesis of a biocompatible polymer through a single-step free radical graft copolymerization of 2-hydroxyethyl methacrylate (HEMA) on soy protein isolate (SPI) to obtain a pH sensitive hydrogel (HEMA-g-SPI) as a potential formulation for targeted drug delivery. It is important to highlight that soy proteins can be efficiently extracted from soybean oil and processed into polymeric hydrogels on an industrial scale at a very low cost. For this reason, this represents a convenient approach for the introduction of an easily obtainable formulation that functions as a drug vehicle. The HEMA-g-SPI hydrogel was developed for the gastrointestinal targeted delivery of paracetamol, a model drug adopted in this study and loaded into the grafted hydrogel. The results indicated that the system is non-cytotoxic, and they also showed differences in drug release based on the pH values of the environment. This element could lead to the use of a protein-based system for the delivery of different classes of poorly water-soluble drugs in areas characterized by harsh conditions, such as the gastrointestinal tract [89].

Soybean protein isolates have been also integrated into polymer nanofibers using an electrospinning technique to enhance the mechanical properties of these fibers. The presence of a big surface area exposed to external substances makes these nanostructures ideal vehicles for drug delivery and targeted release. In this context, an interesting work described the development of PVA (Polyvinyl alcohol)/SPI nanofiber mats and investigated the release of ketoprofen, an anti-inflammatory drug commonly used in various therapies. Sepiolite (a fibrous clay mineral) nanoneedles were incorporated into the polymeric nanofibers to enhance their mechanical features, making them useful for drug loading and delivery. Electrospun nanofiber mats are non-woven fabric-like structures consisting of a network of randomly oriented or aligned nanofibers produced through the electrospinning technique. These mats are characterized by their high surface-area-to-volume ratio, porosity, and small fiber diameter. An investigation of the drug release properties exhibited by the mats was carried out, according to the different formulations developed. The best results were obtained by nanostructures that showed the co-presence of sepiolite needles and PVA, and these mostly related to drug release rate. Further studies are needed to explore this type of formulation in greater depth [90]. 

Zare-Zardini et al. synthetized soybean protein-based nanoparticles (adopting the desolvation technique) in order to evaluate their role for curcumin encapsulation, its loading rate, and its targeted delivery. Moreover, they evaluated antineoplastic activity related to the nanostructures, using osteosarcoma as an example. The outcomes demonstrated that nanoparticles characterized by small dimensions could be adopted as effective systems for slow and controlled drug release. This meant that neoplastic cells were exposed to the anticancer drug for a long period of time; this is a positive result that needs to be validated for different drugs and different therapies while keeping the choice of soybean proteins consistent as the basic component for the building of the nanostructures [91]. 

Wan et al. realized supersaturated nanoemulsions, adopting self-emulsifying techniques [92] with the use of medium chain triglyceride as an oil phase, Tween 80 as a surfactant, and SPI as a raw material, added in the aqueous phase of the nanoemulsion. These nanocompounds were studied for tangeretin (5,6,7,8,4′-pentamethoxyflavone, a natural drug) loading and its controlled release in order to overcome the drug’s poor bioavailability. The nanoencapulation of tangeretin led to an improved release of the drug, so this could represent an effective starting point for the development of nanoemulsion-based delivery vehicles for increasing the bioavailability of hydrophobic pharmaceuticals [93]. 

Quian et al. developed soy protein nanoparticles of different sizes (from 30 to 150 nm) with the adoption of a polymer–monomer pair reaction system, without any organic solvent. The prepared nanoparticles were coated in phenylboronic acid to enhance the nanostructures’ affinity for drug loading. The results showed that the presence of phenylboronic acid enabled the nanocomposites to assume a great targeting affinity for sialic acid, which is overexpressed in many tumor cells. Among all the different sizes, nanoparticles of 30 nm presented the most effective outcomes in studies of cancer cells. This work represents an innovative design strategy for the creation of nanoplatforms for drug delivery in cancer therapeutics [94]. 

All the presented studies are reported in Table 8.

## 4. A Comparative Analysis of Protein-Based Drug Carriers with Other Types of Carriers

**Figure 8:**
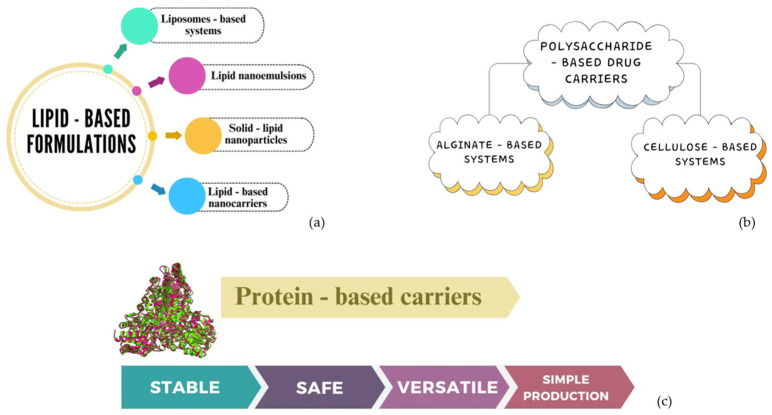
Comparison among lipid-based formulations (**a**), polysaccharide-based drug carriers (**b**), and protein-based carriers (**c**).

Protein-based drug carriers represent a big group of vehicles suitable for effective and controlled drug delivery and targeted release; therefore, it is interesting to focus on the main differences between these systems and several other classes of formulations, as shown in Figure 8.

Starting from lipid-based carriers, lipids are essential constituents of cell membranes; they function as energy storage centers, and they also play an important role in metabolism regulation pathways. The main advantages of lipid-based drug carriers are their high ability to load and protect active substances and to increase their specific release in different areas; on the contrary, the most evident challenges are represented by their unstable structural integrity and drug-releasing properties in different environmental conditions and the exhibition of drug discharge due to the presence of polymorphic transformations [95]. 

Lipid-based formulations include the following:

- liposome-based systems (a challenging aspect is that liposomes could break down and interact with digestive enzymes, so we must focus our research on their stability, release mechanisms, and interactions with the immune system) [96];
- lipid nanoemulsions (the proper choice of lipid types and emulsifiers significantly influences the stability and effectiveness of the carriers) [97];
- solid lipid nanoparticles (which are highly stable and able to provide an effective drug controlled release but, on the other hand, they also present several challenges as drug delivery systems; for example, they have a restricted encapsulation ability for hydrophilic drugs that may represent a limiting factor, considering all the drugs employed in numerous therapies and affected by poor bioavailability) [98];
- lipid-based nanocarriers (with the development of different nanoformulations whose stability can hardly be controlled in harsh environmental conditions) [99].

Another comparison can be made with polysaccharide-based drug carriers, commonly used for biomedical applications because of their favorable features, such as high drug loading efficiency and rapid, controlled, and targeted drug release. This category includes a huge variety of formulations, and here, we provide some examples, such as those based on the use of alginate or cellulose:

- alginate-based drug delivery systems (many carriers have been developed for curcumin delivery but also for the controlled release of tuberculosis drugs; however, a significant challenge with these systems is the physicochemical changes they can undergo in the biological environment, which can alter their drug release capabilities) [100];
- cellulose-based drug delivery systems (based on cellulose’s ability to create compounds with a large surface, they are useful for drugs loading and targeting; different studies show the synthesis of cellulose-containing nanocomposites adopted in anticancer treatments. The main problem with these systems is related to their limited rate of drug controlled release due to changes in the biological environment. Moreover, some polysaccharides show poor mechanical properties and are not compatible with hydrophobic polymers; these disadvantages indicate the need to make surface modifications in order to enhance polysaccharides’ features and use them as effective drug delivery systems) [101].

In this context, protein-based carriers represent a very stable and safe system for efficient drug delivery, more so than the aforementioned systems. Specifically, protein-based nanoparticles can be also incorporated into different polymers for the synthesis of microspheres, for controlled and targeted drug release. Protein nanoparticles offer greater stability and simpler production than other biopolymeric carriers. Moreover, they hold significant promise for in vivo applications, as proteins from various sources can be converted into nanoparticles through straightforward, cost-effective, and environmentally friendly synthesis [102].

## 5. Clinical Development of Protein-Based Drug Delivery Systems 

Clinical trials represent a fundamental part of biomedical research, providing scientific evaluations in order to determine the safety, effectiveness, and possible advantages of new therapies. A clinical study examines the impact of an experimental formulation or any other treatment on a specific group of participants. The research involves a specific group receiving the treatment and a placebo group, with both being assessed to determine the effectiveness of the intervention [103]. The impact of novel pharmaceuticals, medical devices, innovative techniques, and procedures on human participants is closely examined, and the main goal is to produce dependable and impartial information to establish whether a new therapy is secure, successful, and overall better than current alternatives. Carried out in various stages, these studies offer crucial knowledge about medical treatments, aiding in the discovery of elements that can significantly affect how patients respond to treatments, opening the door to tailored medical approaches [104]. Moreover, carefully planned clinical studies, with an in-depth statistical evaluation, are able to offer strong and impartial documentation for the development of new therapeutical approaches. A key aim is to assess if a new treatment is superior to an existing one or if it achieves similar outcomes but is safer, less expensive, or more convenient to adopt [105]. 

Protein-based drug carriers have emerged as a promising avenue in modern medicine, offering advantages such as biocompatibility, specificity, and the ability to be engineered for targeted delivery. Therefore, as the field progresses, the clinical development of these systems is becoming increasingly important, with several key areas of focus shaping the future of this innovative approach.

Below are some examples of clinical studies conducted on carriers prepared using proteins (Table 9).

Tomaya et al. proposed a formulation of cisplatin-loaded gelatin microspheres, and the first clinical outcomes (particularly 1- and 3-month follow-up results of the use of gelatin microparticles of 50 to 100 μm) have shown their advantageous effects on 19 selected patients with advanced hepatocellular carcinoma; every procedure was successfully executed in all participants, and no harmful side effects have been detected [106]. 

An interesting study has described the beneficial role of casein micelles for the nanoencapsulation of vitamin D; the research is focused on the differences in vitamin D bioavailability with the use of casein micelles and synthetic emulsifiers (as Tween 80, which is occasionally adopted by industries to incorporate vitamin D into milk). Ninety healthy adults, aged 18–65 and who passed a medical screening, were randomly divided into three groups, and they received a dietary supplement of different fat-free products: skimmed milk (0% fat) fortified with 50,000 international units of vitamin D in a 150 g product, via a conventional method; skimmed milk with the same amount of vitamin D, emulsified using Tween 80; and placebo: skimmed milk without vitamin D. Blood samples were taken before the products’ consumption and at 1, 7, and 14 days post consumption. The preliminary results show the advantageous aspects of casein micelles’ adoption and their fundamental role in vitamin D-specific and effective delivery [107].

Furthermore, different studies have shown albumin-based nanoparticles involved in anticancer therapies in clinical trials; one example is the nanocomposite Abraxane, adopted as therapeutic agent for pancreatic cancer, non-small cell lung cancer, and breast cancer. In addition, several authors have highlighted the adoption of albumin nanoparticles for the loading and the targeted delivery of Paclitaxel, a chemotherapeutic drug widely used to treat various types of cancer: in a Phase III clinical trial (n° NCT01620190), 503 patients with advanced, previously treated non-small cell lung cancer were randomly assigned to two different groups: 252 patients received albumin NPs–Paclitaxel on days 1, 8, and 15 at a dose of 100 mg/m^2^, while 251 patients received Docetaxel (a commonly administered formulation) at a dose of 60 mg/m^2^ on day 1 of a 21-day cycle. After nearly 3 years of follow-up, serious adverse events such as febrile neutropenia occurred in 2% of the NPs–Paclitaxel group and 22% of the Docetaxel group, while peripheral sensory neuropathy was reported in 10% of the NPs–Paclitaxel group and 1% of the Docetaxel group. All the outcomes showed several advantages related to the use of albumin NPs–Paclitaxel compounds [108]. 

## 6. Conclusions

This review focuses its attention on protein-based drug delivery carriers, which represent a significant advancement in the area of pharmaceutical and biomedical drug targeting and controlled release. These systems employ the unique properties of proteins to improve the delivery and efficacy of therapeutic agents, offering extraordinary benefits for precise and effective treatments related to different diseases. Moreover, numerous modification techniques have been developed in order to provide favorable characteristics to the employed natural proteins, such as optimal particle size, dispersibility, and surface charge. 

Nevertheless, despite these promising attributes, the industrial application of protein-based delivery systems remains limited. To overcome these challenges, upcoming research on protein-based formulations should focus on developing large-scale production methods that allow these vehicles to be manufactured in a commercially feasible way. Moreover, additional scenarios need to be explored in order to leverage all the potential of these fascinating systems. 

Ongoing research and interdisciplinary collaborations are essential to overcome the still existing challenges of these formulations, such as stability, scalability, and targeted delivery, and unlock the full potential of these advanced drug delivery systems. The continuous evolution in this field, driven by technological innovations and deeper biological insights, promises to produce more effective, safe, and patient-friendly therapeutic options in the near future. While achieving ideal protein-based drug carriers remains a significant challenge, ensuring biocompatibility and enhancing in vivo performance should remain top priorities.

Finally, the integration of novel materials and techniques is expected to lead to significant breakthroughs, further enhancing the capabilities and applications of protein-based drug delivery systems. 

## Figures and Tables

**Table 1:** Key differences between protein-based biopolymers and synthetic polymers.

Characteristic	Protein-Based Biopolymers	Synthetic Polymers
Water solubility	High	Varies, generally low
Biocompatibility	High	Varies, generally low
Biodegradability	High	Generally low
Toxicity	Low	Potentially high
Inertness	Inert	Potentially reactive
Availability	Easily available from natural sources	Depends on the synthesis process
Amphiphilic properties	Yes, facilitating interactions with solvents and drugs	Generally lacking
Conjugation abilities	Capable of forming covalent bonds with drugs and ligands	Good conjugation abilities but with potential side effects
Role in immune response	Less likely to cause immune response activation	Can cause inflammation and immune response activation
Use of organic solvents	Not required	Often required

**Table 2:** Gelatin-based drug delivery systems.

Protein-Based Carrier	Drug	Application	Reference
Cassava starch acetate (CSA)—polyethylene glycol (PEG)—gelatin (G) nanocomposites	Cisplatin	Anticancer therapy	[33]
Eudragit-S100-coated gelatin nanoparticles	5-amino salicylic acid (5-ASA)	Ulcerative colitis treatment	[34]
Graphene oxide nanocarriers covered by gelatin and polyvinylpyrrolidone (PVP)	Quercetin	Anticancer therapy	[35]
Carbon quantum dots complexed with gelatin and chitosan hydrogel	Curcumin	Anticancer therapy	[36]
Halloysite nanotubes complexed with gelatin microparticles	Carvedilol	Improved oral drug delivery system for hypertension and coronary artery pathologies	[37]
Gelatin nanoparticles	Methotrexate	Anticancer therapy	[39]
Insoluble gelatin type B/chitosan nanoparticles	Systems tested as good Pickering emulsifiers	Numerous different treatments	[42]
Gelatin/glucomannan)/tannic acid nanocomplexes	Systems tested as good Pickering emulsifiers	Numerous different treatments	[43]
A multilayer emulsion made up of gelatin, gum Arabic and tannic acid	Curcumin	Anticancer therapy	[44]
Aminated gelatin nanoparticles	Systems tested as good Pickering emulsifiers	Numerous different treatments	[46]
Gelatin nanoparticles	Tizanidine hydrochloride and Gatifloxacin	Muscle relaxation therapies and bacterial infection treatments, also anticancer therapy	[47]
Gelatin/folic acid nanoparticles	Irinotecan	Anticancer therapy	[48]
Gelatin nanoparticles of different sizes	Doxorubicin, iodixanol and cisplatin	Anticancer therapy	[49]

**Table 3:** Albumin-based drug delivery systems.

Protein-Based Carrier	Drug	Application	Reference
BSA/oxidized arabic gum nanoparticles	Piperine	Anticancer therapy	[51]
Folic acid–BSA grafted graphene oxide nanocomplexes	Doxorubicin	Anticancer therapy	[52]
Ethoniosomes coated with folic acid/BSA	Pterostilbene	Antidiabetic and anticancer therapies	[55]
Fe^3+^–BSA nanoparticles, grafted with folic acid and complexed with indocyanine green dye	Doxorubicin	Anticancer therapy	[56]

**Table 4:** Collagen-based drug delivery systems.

Protein-Based Carrier	Drug	Application	Reference
Collagen (poly 3-acrylamidophenylboronic acid, PAPBA) nanoparticles	Doxorubicin	Anticancer therapy	[57]
Type 1 collagen hydrogels	Luteolin	Wound-healing therapies	[58]
An innovative nanostructure made up of cellulose nanofibrils and collagen aerogels	5-fluorouracil	Numerous different treatments	[59]
Porous microspheres made up of collagen and bacterial cellulose	BSA	Numerous different treatments	[60]
Collagen nanoparticles	Sylimarin	Brain disease therapies	[61]

**Table 5:** Zein-based drug delivery systems.

Protein-Based Carrier	Drug	Application	Reference
Zein nanoparticles (phase separation method)	Doxorubicin	Anticancer therapy	[63]
Zein nanoparticles	Maytansine	Anticancer therapy	[64]
Zein nanoparticles coated with sodium caseinate	Luteolin	Wound-healing therapies	[65]
Zein nanoparticles	PTEN (Phosphatase and Tensin homolog deleted from chromosome ten) and TRAIL (TNF- related apoptosis- inducing ligand) genes	Gene therapy and anticancer therapy	[66]
Zein nanofibers made up of chitosan and polyethylene oxide (PEO)	Alpha-tocopherol	Delivery of hydrophobic compounds to the gastrointestinal area	[68]
Zein nanofibers with the incorporation of tungsten oxide	Tested as innovative structures	Anticancer therapy	[69]

**Table 6:** Gliadin-based drug delivery systems.

Protein-Based Carrier	Drug	Application	Reference
Gliadin nanoparticles coated by polyoxyethylene (2) oleyl ether	Doxorubicin hydrochloride	Anticancer therapy	[71]
Gliadin nanoparticles functionalised with hyaluronic acid	Usnic acid	Anticancer therapy	[72]
Hybrid gliadin/silver nanoparticles for the building of an innovative protein-based porous material	Tested as innovative structures	Antibacterial therapies	[73]
Nanomicelles made up of gliadin hydrolysates	Naringin	Anticancer therapy	[74]
Gliadin nanoparticles coated by caseins	Amphotericin B	Antifungal infections treatments	[75]

**Table 7:** Silk protein-based drug delivery systems.

Protein-Based Carrier	Drug	Application	Reference
Silk fibroin nanoparticles	Quercetin	Anticancer and anti-inflammatory therapies	[77]
Silk fibroin–chitosan nanoparticles	Curcumin	Anticancer therapy	[78]
Silk fibroin/casein electrospun nanofibers	Diclofenac sodium salt	Anti-inflammatory therapies	[79]
Silk fibroin–human serum albumin nanocapsules	Methotrexate	Anti-inflammatory therapies	[80]
Silk sericin nanoparticles	Atorvastatin	Anticancer therapy	[84]
Silk sericin nanoparticles covered by pluronic F-68	Resveratrol	Anticancer therapy	[85]
Silk sericin/poly(ethylcyanoacrylate) nanospheres	Fenoifibrate	Cholesterolemia therapies	[86]
Bioconjugate made of silk sericin with modifications	Sunitib	Anticancer therapy	[87]

**Table 8:** Soybean protein-based drug delivery systems.

Protein-Based Carrier	Drug	Application	Reference
2-hydroxyethyl methacrylate—soy protein isolate (SPI) pH sensitive hydrogel	Paracetamol	Gatrointestinal disease therapies	[89]
PVA (Polyvinyl alcohol)/SPI nanofiber mats complexed with sepiolite nano needles	Ketoprofen	Anti-inflammatory therapies	[90]
Soybean protein-based nanoparticles	Curcumin	Anticancer therapy	[91]
Supersaturated nanoemulsions of SPI	Tangeretin	Numerous therapies involving hydrophobic pharmaceuticals	[93]
Soy protein nanoparticles coated by phenylboronic acid	Sialic acid	Anticancer therapy	[94]

**Table 9:** Clinical studies on protein-based drug delivery systems.

Protein-Based Carrier	Drug	Application	Reference
Gelatin nanoparticles	Cisplatin	Clinical trial first stage—Advanced Hepatocellular Carcinoma	[86]
Casein micelles	Vitamin D	Clinical trial n° NCT01807845	[87]
Albumin nanoparticles	Paclitaxel	Clinical trial n° NCT01620190	[88]
